# Impact of Comorbidities on Hospitalization for Injuries in Riders of Electric Bikes and Powered Scooters: A Retrospective Cross-Sectional Study

**DOI:** 10.3390/medicina58050659

**Published:** 2022-05-13

**Authors:** Yafit Hamzani, Helena Demetriou, Adi Zelnik, Nir Cohen, Michael J. Drescher, Gavriel Chaushu, Bahaa Haj Yahya

**Affiliations:** 1 Department of Oral and Maxillofacial Surgery, Rabin Medical Center—Beilinson Hospital, Petach Tikva 4941492, Israel; drlilidemetriou@gmail.com (H.D.); gabi.chaushu@gmail.com (G.C.); 2 Maccabi-Dent, Holon 5810001, Israel; adi.zelnik@gmail.com; 3 Department of Orthopedic Surgery, Rabin Medical Center—Beilinson Hospital, Petach Tikva 4941492, Israel; nirco2@clalit.org.il; 4 Sackler Faculty of Medicine, Tel Aviv University, Tel Aviv 6997801, Israel; michaeldr@clalit.org.il; 5 Department of Emergency Medicine, Rabin Medical Center—Beilinson Hospital, Petach Tikva 4941492, Israel; 6 The Maurice and Gabriela Goldschleger School of Dental Medicine, Tel Aviv University, Tel Aviv 6997801, Israel; 7 Oral and Maxillofacial Private Clinic, Herzliya 4685107, Israel; bahaa.hag@gmail.com

**Keywords:** emergency department, medical condition, medications, electric bikes, powered scooters, injury

## Abstract

*Background and Objectives:* Injuries associated with electric bikes (E-bikes) and powered scooters (P-scooters) have increased yearly worldwide. We aimed to evaluate the impact of pre-existing comorbidities on the probability of hospitalization for injuries in riders of E-bikes and P-scooters. *Materials and Methods:* A retrospective cross-sectional study design was used. The cohort included patients referred to the emergency department (ED) of a tertiary medical center in 2014–2020 for injuries sustained while riding an E-bike or P-scooter. Data were collected from the medical files on demographics, clinical characteristics including pre-existing comorbidities and permanent use of medications, and injury characteristics. Findings were compared between patients referred for hospitalization from the ED and patients discharged home. *Results*: Of the 1234 patients who met the inclusion criteria, 202 (16.4%) had a prior medical condition and 167 (13.5%) were taking medication on a permanent basis. A significant relationship was found between hospitalization and having a medical condition (𝜒^2^(1) = 9.20, *p* = 0.002) or taking medication on a permanent basis (𝜒^2^(1) = 6.24, *p* = 0.01). Hospitalization for injuries was more likely in patients with a comorbidity (27.8%) than those without a comorbidity (15.5%), and in patients who were on permanent drug therapy (22.2%) than in patients who were not (12.9%). Surprisingly, anticoagulant intake specifically had no effect on the probability of hospital admission. *Conclusions:* Patients with comorbidities have a higher incidence of hospitalization for E-bike- and P-scooter-associated injuries. Therefore, physicians may take into account comorbidities for the effective management of this patient group’s injuries.

## 1. Introduction

The increasing use of electric bicycles (E-bikes) and powered scooters (P-scooters) in recent years has been accompanied by associated injuries [1,2]. Studies of the characteristics of riders injured in E-bike and P-scooter accidents have shown that the majority were healthy males in their 30s [1,2,3,4,5,6,7,8,9,10,11].

Electric vehicle injuries mainly occur in the extremities and maxillofacial areas [2,11]. A recent study aiming to assess the frequency and severity of maxillofacial injuries associated with electric vehicles in Israel between the years 2014 and 2019 found that 10.3% out of a total of 3686 hospital admissions were oral and maxillofacial injuries. Out of those, 37.4% were elders older than 60 years [12].

It is widely recognized that advanced age and comorbidities have a crucial impact on the probability of requiring medical care and hospital admission in cases of injury [13]. Moreover, the intake of medication, and particularly antiplatelet agents, as a solitary factor can increase this risk [14]. The widespread use of direct oral anticoagulants has outpaced research efforts to establish their effects in patients with bleeding traumatic injuries [15]. Most of the available studies focused on intracranial hemorrhage without assessing the need for hospital admission [13,14,15,16].

The aim of the present study was to evaluate the impact of pre-existing comorbidities and the permanent use of medication, with an emphasis on anticoagulants, on the rates of hospitalization for injuries sustained by riders of E-bikes and P-scooters.

## 2. Materials and Methods

A retrospective, cross-sectional study was conducted in the emergency department (ED) of a tertiary medical center from January 2014 to March 2020. ED referrals of patients that were injured while riding P-scooters and E-bikes met the inclusion criteria of the study. The ED database was first searched using the keywords “electric scooter” or/and “electric bike” and/or “powered scooter” or/and “powered bike” and “injury/injured”. The files of the patients identified were then further reviewed, and those who were found to have been actually involved in an E-bike or P-scooter accident during the study period and for whom sufficient data were available were included in the study.

The following data were collected from the medical files: demographics, comorbidities, intake of permanent medications, use and specific type of anticoagulants, reason for prescription of anticoagulants, bleeding/hemorrhage, bleeding organs, need for hospitalization for current E-bike or P-scooter injury, and length of current hospitalization. The findings were compared between patients who were hospitalized and patients who were discharged home from the ED. An injury requiring hospitalization was defined as severe. The study protocol was approved by the Helsinki Committee of Rabin Medical Center (approval number 0194-20-RMC).

The data were analyzed using SPSS statistical software, version 25 (IBM, Armonk, NY, USA). Continuous variables were summarized by means and standard deviations and discrete variables were depicted as frequencies. Chi-square test was used for univariate analysis. Owing to the non-normal distribution of the continuous variables, the nonparametric Mann–Whitney test was used to compare independent samples. Significance was defined as a *p*-value lower than 5%.

## 3. Results

A total of 1234 patients met the inclusion criteria; all of the patients were referred to the ED of “Rabin medical center” in Petah Tikvah, Israel regarding injuries associated with E-bikes or P-scooters during the study period. The full descriptive statistics of the cohort are shown in Table 1 and Table 2. There were 934 male (75.7%) and 300 female (24.3%) patients of mean age 31.52 (±14.77) years. Ninety patients (7.3%) suffered a severe injury and thus required hospitalization. The mean number of admission days was 5.44 (±0.12). A known medical condition was documented in 202 patients (16.4%). Diabetes was the most common comorbidity, observed in 36 patients (2.9%).

Permanent medications were being used by 167 patients (13.5%). Medications for diabetes were the most common prescribed (*n* = 30, 2.4% of the cohort). Eighteen patients (1.46%) reported being on permanent anticoagulant therapy. Aspirin, a salicylate antithrombotic agent, was the most common anticoagulant used (*n* = 9, 0.7% of the cohort).

The E-bike/P-scooter injury was associated with hemorrhaging in 131 patients (10.6%) and with a bleeding organ in 124 patients (10.1%). The most common sites of bleeding were the eyes; intra-bulbar hemorrhage and the surrounding soft tissue, including the eyelids, in 28 patients (2.3%).

Univariate analyses were used to test the association of each independent variable with hospitalization for injuries associated with E-bikes and P-scooters. The results are shown in Table 3. A significant relationship was found between the presence of a medical condition and hospitalization (𝜒^2^(1) = 9.20, 𝑝 = 0.002). Patients with comorbidities were more likely to be hospitalized (27.8%) than patients without comorbidities (15.5%). There was also a direct relationship between intake of permanent medications and hospitalization (𝜒^2^(1) = 6.24, 𝑝 = 0.01), with patients who were on permanent drug therapy having a higher likelihood of hospitalization (22.2%) than patients who were not (12.9%).

Hospitalization was significantly associated with hemorrhaging or bleeding (𝜒^2^(1) = 11.22, *p* = 0.001) and with bleeding from an organ (𝜒^2^(1) = (1) = 13.38, *p* = 0.001). Patients with hemorrhaging or bleeding were more likely to be hospitalized (21.1%) than patients without hemorrhaging or bleeding (9.8%), as were patients with a bleeding organ (21.1%) compared to patients without a bleeding organ (9.8%). The use of anticoagulants was not associated with an increased incidence of hospital admission.

## 4. Discussion

We investigated whether the presence of comorbidities or the use of medications on a permanent basis in riders of E-bikes and P-scooters impacted the severity of injuries they sustained in accidents involving these vehicles, as measured by the likelihood of hospitalization compared to discharge from the ED.

Analysis of the clinical characteristics of our cohort revealed a predominance of healthy male patients in the fourth decade of life, similar to other relevant studies in the literature [3,6,7,8,9,10].

Long-term medications are used by individuals with multiple comorbidities and the elderly. They include anticoagulants, which are prescribed to prevent ischemic stroke in patients with atrial fibrillation and to treat acute venous thromboembolism [15]. Anticoagulants can be classified by their mode of action into three main types: vitamin K antagonists (VKA), direct oral anticoagulants, and low-molecular-weight heparin [15].

In the medically compromised population, impaired mobility, sight, and stability often lead to injuries due to falls and traffic accidents. On the basis of studies describing the impact of anticoagulant use on brain injuries [13,14,15,16], we assumed that riders of E-bikes and P-scooters who used anticoagulants would have higher rates of morbidity and hospitalization than riders who did not [13,14,15,16]. However, the results show no such effect of anticoagulant use. This finding may be attributable to the small proportion of patients who reported using anticoagulants (*n* = 18, 1.46%) relative to the number reporting use of any prior permanent medications (*n* = 167, 13.5%). Moreover, our evaluation of the direct impact of anticoagulant use on head injuries was limited because of a lack of data on head injuries specifically and helmet use.

Diabetes mellitus is a widespread chronic disease that leads to impaired wound healing and can be classified as type 1 or type 2 [17,18]. Type 1, or insulin-dependent diabetes, is an autoimmune disease characterized by the destruction of insulin-producing pancreatic β cells in the islets of Langerhans leading to insulin deficiency [19]. Type 2, or non-insulin-dependent diabetes, the more common type, is characterized by increased plasma glucose levels due to insulin secretion deficiencies and insulin resistance [18].

At the cellular level, hyperglycemia interferes with wound healing owing to its adverse effects on neutrophil, lymphocyte, and mast cell function. It hampers red blood cell permeability and decreases blood flow through small vessels at wound surfaces resulting in impaired chemotaxis and phagocytosis [20]. A recent study showed that the function of skin mast cells is impaired because of diabetes-induced degranulation^17^. Furthermore, hyperglycemia causes the delayed release of oxygen from the hemoglobin complex, leading to oxygen and nutrient deficiency in the wound area [20].

At the tissue level, studies found diabetes to be significantly associated with an increased risk of insufficient hard-tissue [21] and soft-tissue healing [22]. In our cohort of E-bike and P-scooter riders, diabetes mellitus was the most common comorbidity (*n* = 36; 2.9%), and diabetic medications were the most common permanent drugs used (*n* = 30; 2.4%). We assume that the impaired wound healing due to diabetes results in hard- and soft-tissue wound complications. As a consequence, the injuries sustained are more severe and require in-hospital management. This is supported by the significant relationship found here between having a medical condition and hospitalization (*p* = *0*.002).

## 5. Conclusions

In conclusion, the present study shows that the presence of underlying comorbidities and the long-term intake of medications increase the probability of hospitalization for injuries in E-bike and P-scooter accidents. The medical staff of the ED should be alert to the need to take the presence of comorbidities and permanent medication intake into account in predicting the severity of injury and the management of this patient group, as reported in the current study as a predictor for hospital admission.

## Figures and Tables

**Table 1:** Demographic information of 1234 injured riders of E-bikes and P-scooters.

Characteristic	Value
**Sex**	
Male	934 (75.69%)
Female	300 (24.31%)
**Age**	31.52 ± 14.77
**Medical condition**	
Healthy	1032 (83.63%)
Diabetes	36 (2.9%2)
Psychiatric disease	29 (2.35%)
Hypertension	23 (1.86%)
Smoker	15 (1.22%)
Cardiovascular disease	9 (0.73%)
Obesity	7 (0.57%)
Dyslipidemia	6 (0.49%)
Cancer	5 (0.41%)
Renal failure/insufficiency	3 (0.24%)
Osteoporosis	2 (0.16%)
Epilepsy	2 (0.16%)
Hypothyroidism	1 (0.08%)
Cystic fibrosis	1 (0.08%)
Other	134 (10.86%)
**Regular medication**	
None	1067 (86.47%)
Diabetes medications	30 (2.43%)
Antihypertensive medications	27 (2.19%)
Psychiatric medications	23 (1.86%)
Anticoagulants	18 (1.46%)
Statins	12 (0.97%)
Cancer medications	2 (0.16%)
Epileptic medications	2 (0.16%)
Bisphosphonates	2 (0.16%)
Steroids	1 (0.08%)
Hypothyroidism medications	1 (0.08%)
Other	103 (8.35%)
**Type of anticoagulant**	
None	1216 (98.5%)
Aspirin	9 (0.73%)
Plavix	4 (0.32%)
Eliquis	2 (0.16%)
Xarelto	2 (0.16%)
Coumadin	1 (0.08%)

**Table 2:** Hemorrhaging and hospitalization of 1234 injured riders of E-bikes and P-scooters.

Characteristic	Value
**Bleeding/hemorrhage**	131 (10.6%)
**Bleeding organ**	
None	1110 (89.95%)
Nose (epistaxis)	12 (0.97%)
Lips	14 (1.13%)
Chin	18 (1.46%)
Intra Cranial	17 (1.38%)
Upper limbs	17 (1.38%)
Lower limbs	15 (1.22%)
Cheek	4 (0.32%)
Intrabulbar and surrounding soft tissue	28 (2.27%)
Stomach	6 (0.49%)
Ear	2 (0.16%)
**Hospital admission**	
No	1144 (92.71%)
Yes	90 (7.29%)

**Table 3:** Univariate tests of association of all variables with hospitalization.

Variable	Discharged	Hospitalized	*p*
**Sex**			
Male	855 (74.8%)	78 (86.7%)	0.12
Female	288 (25.2%)	12 (13.3%)	
**Medical condition**			
No	966 (84.5%)	65 (72.2%)	0.002
Yes	177 (15.5%)	25 (27.8%)	
**Regular medications**			
No	996 (87.1%)	70 (77.8%)	0.01
Yes	147 (12.9%)	20 (22.2%)	
**Type of anticoagulant**			
None	1128 (98.6%)	88 (97.8%)	0.60
Aspirin	7 (0.6%)	2 (2.2%)	
Plavix	4 (0.3%)	0 (0%)	
Coumadin	1 (0.1%)	0 (0%)	
Eliquis	2 (0.2%)	0 (0%)	
Xarelto	2 (0.2%)	0 (0%)	
**Bleeding/hemorrhage**			
No	1030 (90.2%)	71 (78.9%)	0.001
Yes	112 (9.8%)	19 (21.1%)	
**Bleeding organ**			
No	1038 (90.9%)	71 (78.9%)	<0.001
Yes	104 (9.1%)	19 (21.1%)	

## Data Availability

The authors confirm that the data supporting the findings of this study are available within the article.
